# Systemic inflammatory response and neuromuscular involvement in amyotrophic lateral sclerosis

**DOI:** 10.1212/NXI.0000000000000244

**Published:** 2016-06-01

**Authors:** Ching-Hua Lu, Kezia Allen, Felicia Oei, Emanuela Leoni, Jens Kuhle, Timothy Tree, Pietro Fratta, Nikhil Sharma, Katie Sidle, Robin Howard, Richard Orrell, Mark Fish, Linda Greensmith, Neil Pearce, Valentina Gallo, Andrea Malaspina

**Affiliations:** From the Centre for Neuroscience & Trauma (C.L., F.O., J.K., A.M.) and Centre of Primary Care and Public Health (V.G.), Blizard Institute, Barts and The London School of Medicine and Dentistry, Queen Mary University of London; Sobell Department of Motor Neuroscience and Movement Disorders (C.L., P.F., L.G.), MRC Centre for Neuromuscular Diseases (P.F., L.G.), MRC Unit for Lifelong Health and Ageing (N.S.), Department of Molecular Neuroscience (K.S.), and Department of Clinical Neuroscience (R.O.), UCL Institute of Neurology; Basildon and Thurrock University Hospitals (K.A., A.M.), NHS Foundation Trust, Basildon; William Harvey Hospital (F.O.), Kent; Proteome Sciences plc (E.L.), South Wing Laboratory, Institute of Psychiatry, UK; Neurology (J.K.), University Hospital Basel, Switzerland; Department of Immunobiology (T.T.), King's College London; National Hospital for Neurology and Neurosurgery (N.S., R.H., R.O.), London; Musgrove Park Hospital (M.F.), Taunton; and Department of Medical Statistics (N.P.), London School of Hygiene and Tropical Medicine, UK.

## Abstract

**Objective:**

To evaluate the combined blood expression of neuromuscular and inflammatory biomarkers as predictors of disease progression and prognosis in amyotrophic lateral sclerosis (ALS).

**Methods:**

Logistic regression adjusted for markers of the systemic inflammatory state and principal component analysis were carried out on plasma levels of creatine kinase (CK), ferritin, and 11 cytokines measured in 95 patients with ALS and 88 healthy controls. Levels of circulating biomarkers were used to study survival by Cox regression analysis and correlated with disease progression and neurofilament light chain (NfL) levels available from a previous study. Cytokines expression was also tested in blood samples longitudinally collected for up to 4 years from 59 patients with ALS.

**Results:**

Significantly higher levels of CK, ferritin, tumor necrosis factor (TNF)–α, and interleukin (IL)–1β, IL-2, IL-8, IL-12p70, IL-4, IL-5, IL-10, and IL-13 and lower levels of interferon (IFN)–γ were found in plasma samples from patients with ALS compared to controls. IL-6, TNF-α, and IFN-γ were the most highly regulated markers when all explanatory variables were jointly analyzed. High ferritin and IL-2 levels were predictors of poor survival. IL-5 levels were positively correlated with CK, as was TNF-α with NfL. IL-6 was strongly associated with CRP levels and was the only marker showing increasing expression towards end-stage disease in the longitudinal analysis.

**Conclusions:**

Neuromuscular pathology in ALS involves the systemic regulation of inflammatory markers mostly active on T-cell immune responses. Disease stratification based on the prognostic value of circulating inflammatory markers could improve clinical trials design in ALS.

Clinical heterogeneity in amyotrophic lateral sclerosis (ALS), a fatal neurodegenerative disorder, encompasses motor and cognitive symptoms and a variable prognostic outlook. The long diagnostic latency in most ALS cases narrows the therapeutic window for disease modification. Early diagnosis and the ability to predict outcomes in ALS would address the unsatisfactory outcome of most clinical trials with the design of more cost-effective studies.^1^ Current biomarkers-based monitoring tools in ALS are not always suitable for research and routine clinical practice.

The pathologic process in ALS develops in distant anatomical regions either simultaneously or as a sequential process.^2^ Early ALS pathology is sensed by the innate immune system, with the activation of microglia, T-cells, dendritic, and antigen-presenting cells in corticospinal tracts and in the motor cortex^3^ and the release of inflammatory markers such as cytokines, C-reactive protein (CRP), and ferritin.^4–6^

Circulating inflammatory markers and immune cells express the body's inflammatory state, which depends on comorbidities and environmental stressors. The immunologic fingerprint of ALS at a systemic level may not be easily distinguishable, considering the reported strong association of ALS with autoimmune comorbidities such as rheumatoid arthritis.^7^ Nevertheless, ALS-specific systemic inflammatory signals have already been reported,^4–6,8^ including a reduced frequency of regulatory T cells (Treg) in blood from individuals with a faster disease progression.^9^

By adjusting for potential contributors to systemic inflammation,^4^ we tested to what extent the expression of circulating markers of inflammation and of neuromuscular pathology changes in ALS with reference to control individuals; we have used the same approach for the prognostic stratification of ALS and to test the systemic immune response to disease progression.

## METHODS

### Standard protocol approvals, registrations, and patient consents.

Ethical approval was obtained from the East London and the City Research Ethics Committee 1 (09/H0703/27). All participants provided written consent (or gave verbal permission for a carer to sign on their behalf).

### Participants, sampling, and data collection.

The study included 95 patients with ALS and 88 neurologically healthy controls recruited between 2009 and 2015. Diagnosis of ALS by experienced ALS neurologists (A.M., R.H., R.O., K.S., P.F., N.S., M.F.) was based on consensus criteria.^10^ Those with a family history of ALS or frontotemporal dementia (FTD), or known to carry a genetic mutation linked to ALS or FTD, were excluded to minimize any potential bias. Neurologically healthy controls were typically spouses and friends of patients.

Serial plasma samples and clinical information were obtained, on average, every 2–4 months from 59 of the 95 patients with ALS, over a maximum follow-up period of 48 months. No selection criteria were applied to patients with ALS sampled longitudinally, other than their willingness to donate further samples. Symptoms onset was defined as first patient-reported weakness or speech impairment. Progression rate was calculated at baseline (PRB) as 48 minus the ALS Functional Rating Scale–Revised (ALSFRS-R) score, divided by the disease duration from symptom onset in months. A PRB of 0.5 was used as cutoff to define slowly progressing (<0.5) and fast progressing ALS (≥0.5). Progression between 2 consecutive visits was assessed using the change in ALSFRS-R score (ΔALSFRS-R) with and without the time interval (ΔALSFRS-R/duration between 2 visits in months).^11^

Data including demographics, medical history, and treatment were collected at each visit. The presence of hypertension, hyperlipidemia, diabetes, cancer, and cardiac and cerebrovascular accidents as well as prothrombotic states were systematically evaluated at each visit. QRISK2, a cerebrovascular disease risk score, was included in the statistical analyses.^12^

### Sample analysis.

#### Cytokines.

Plasma samples were processed and aliquoted within 1 hour from collection and frozen at −80°C, following standard consensus procedures.^13^ A validated multiplex electrochemiluminescence immunoassay was used for the analysis of interferon (IFN)–γ, tumor necrosis factor (TNF)–α, interleukin (IL)–6, IL-1β, IL-2, IL-8, IL-12p70, IL-4, IL-5, IL-10, and IL-13 from patients with ALS and controls in duplicate (Meso Scale Discovery, Rockville, MD). Readouts in the multiplex assay with poor intra-assay coefficient of variation (>20%) were excluded from further analyses.

#### CRP, creatine kinase (CK), and ferritin.

CRP, ferritin, and CK were measured only at baseline and following the guidelines from the International Federation of Clinical Chemistry. CRP and CK were tested using an immunoturbidimetric assay on a Roche/Cobas (Indianapolis, IN) 702 module. CRP readouts below the laboratory dynamic range were given the lower limit value of 2 mg/L. Ferritin was tested on a Roche/Cobas e602 module, using the Roche ferritin electrochemiluminescence immunoassay.

### Statistical analysis.

Continuous variables were summarized using median (interquartile range [IQR]) and their distribution tested using Mann-Whitney *U* test. Distribution of categorical data was tested using the Fisher exact test.

Baseline analysis of the case-control study included (1) univariate comparison using the Mann-Whitney *U* test, (2) principal component analysis (PCA) to test combined cytokine effects (log2 transformed data were analyzed using TIGR MeV, TM4, version 4.9), and (3) multivariate logistic regression analysis. Tertiles of distribution of continuous variables calculated on the whole sample^14^ were introduced in logistic regression models (table e-1 at Neurology.org/nn). Cytokine data with undetectable levels were assigned the midpoint between zero and the limit of detection (LOD; provided by the manufacturer).^15^ When more than 33% of the samples had measurements below the LOD, all undetectable samples were included in the lowest tertile, while the median of the remaining samples defined the second cutoff point. Logistic regressions included a basic model adjusted for age and sex, a multivariate model adjusted for comorbidities,^7,16^ and a mutually adjusted model where all the inflammatory markers associated with the outcome with *p* < 0.2 were simultaneously included (either in a basic or in a multivariate model). Comorbidities included in multivariate models are arthritis, autoimmune pathology, hypertension, diabetes, hyperlipidemia, the use of statin, cardiovascular disease (CVD) risk, and CRP as proxy for general inflammation. This latter mutually adjusted analysis was undertaken excluding subjects with missing data and using redefined tertile ranges (table e-1). Logistic regression was also applied to compare progression rate groups among ALS cases at baseline; tertile ranges were redefined within these ALS cases only (table e-1).

Survival analysis was conducted using Cox regression with tertiles recalculated from ALS cases only. The basic and multivariate statistical models (defined as above) were used to test to what extent each plasma marker predicted survival individually. Basic and multivariate mutually adjusted models were also run.

Correlation between continuous variables was assessed using the Spearman coefficient (ρ), which measures the strength of a monotonic relationship between paired data and varies between −1 (perfect monotonic negative correlation) and 1 (perfect monotonic positive correlation). We examined the association between plasma cytokines and disease progression/stage as well as biological markers of (1) muscle homeostasis (CK), (2) neuroaxonal damage (neurofilament light chain [NfL]; baseline data from 66 out of 95 of the patients with ALS were available from a previous study^17^), and (3) other inflammatory markers (ferritin and CRP).

Data collected from the 59 patients with ALS serially sampled were used for the longitudinal analysis of the 11-cytokines panel. A Kruskal-Wallis test was used to compare expression data obtained at baseline (V1) and at follow-up visits (V2–V6). Intervisit progression (e.g., V1 and V2, V2, and V3), as described in a previous study,^11^ was defined as (1) the change of ALSFRS-R score between visits (ΔALSFRS-R) and (2) the slope of ALSFRS-R (ΔALSFRS-R/time between 2 visits in months). The cytokine levels at early visit (in each visit pair) were correlated with the intervisit progression calculated from the same visit pair (Spearman). Intervisit progression for CRP, CK, and ferritin was calculated only between V1 and V2.

Statistical analysis was performed using SPSS (version 22; IBM, Armonk, NY) and GraphPad Prism Software (GraphPad Software, La Jolla, CA; version 6). Unless otherwise specified, a *p* value less than 0.05 was considered statistically significant.

## RESULTS

### Case-control analysis.

Medical history, demographic data, and levels of the plasma markers are presented in tables 1 and 2. Patients with ALS were older and predominantly male compared to controls; hyperlipidemia was more prevalent in controls. Patients with ALS had a higher CVD risk.

**Table 1.**
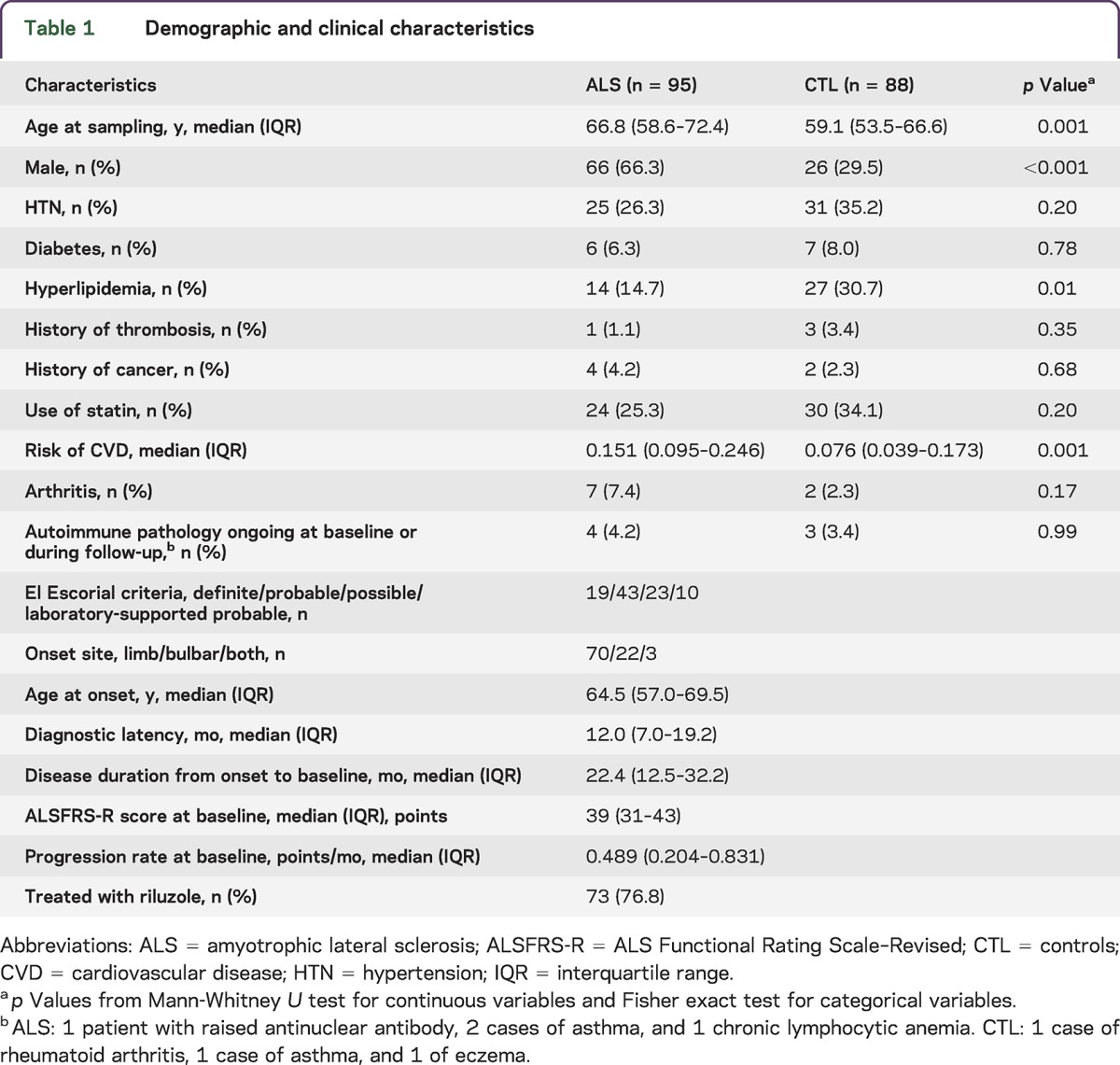
Demographic and clinical characteristics

**Table 2.**
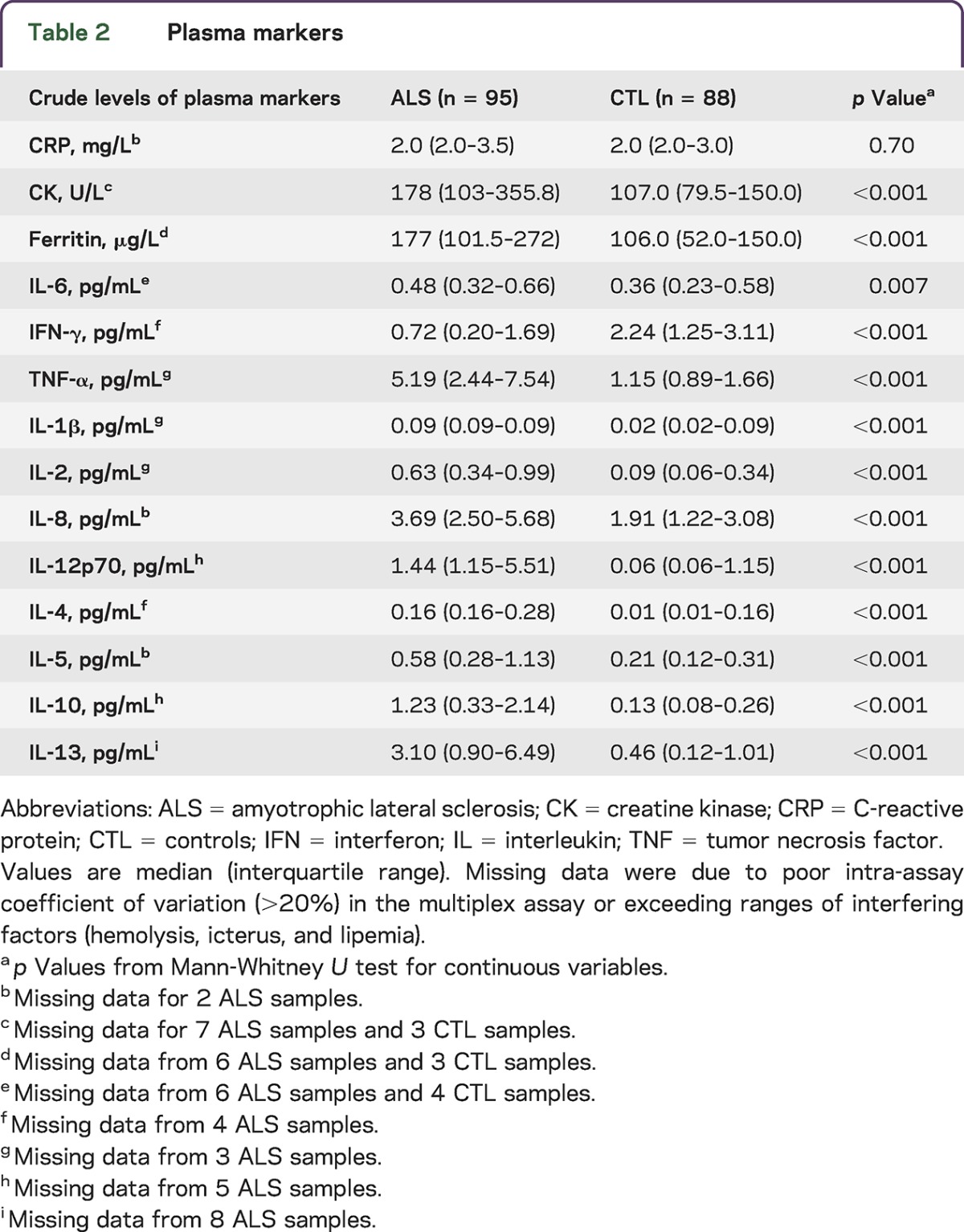
Plasma markers

**Figure 1.**
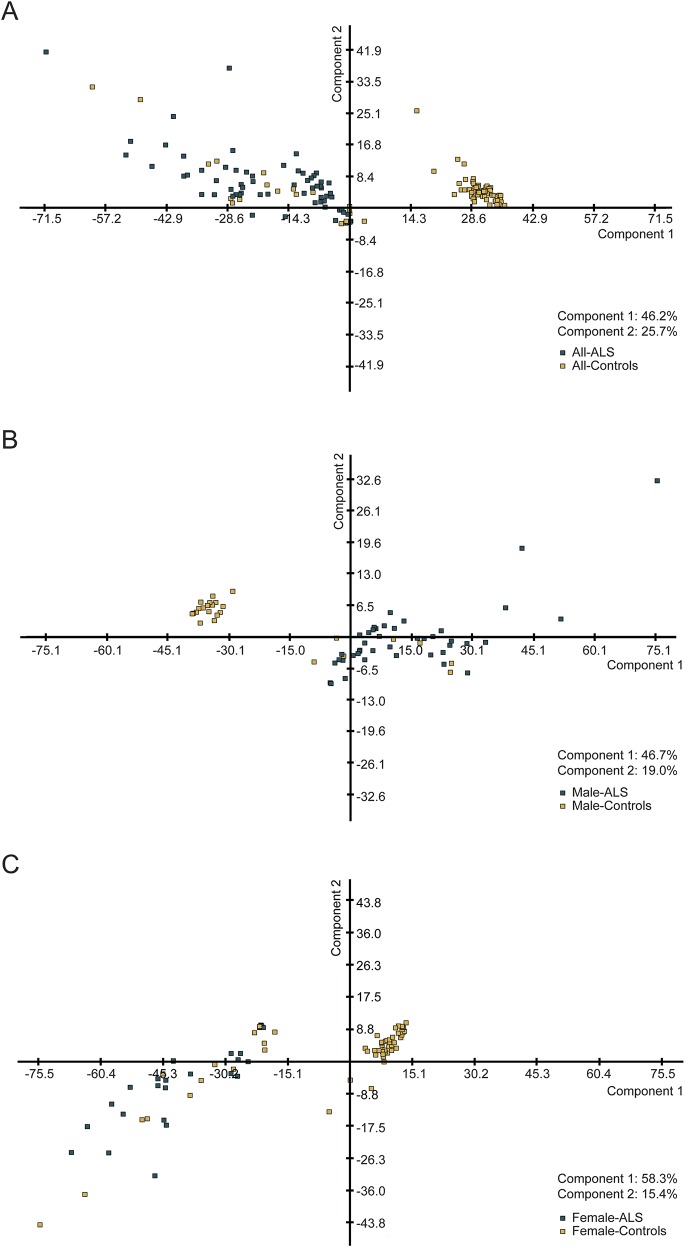
Principal component analysis (PCA) of patients with amyotrophic lateral sclerosis (ALS) vs controls PCA analysis shows a good separation between ALS and controls of the expression levels of the 11 cytokines under investigation, more significant when sex-specific groups are considered. (A) ALS (green squares) and controls (yellow squares), (B) male only ALS (green squares) and controls (yellow squares), and (C) female only ALS (green squares) and controls (yellow squares).

PCA showed a good separation between ALS and control groups with regard to cytokine expression profiles (component 1: 46.2%; component 2: 25.7%; figure 1A). A better separation was obtained when the data were grouped by sex, particularly in the female group (component 1: 58.3%; figure 1, B and C). There was no meaningful separation between ALS subgroups when categorized according to site of disease onset, progression rate at baseline, and disease stages.

### Regression analysis.

#### Logistic regression analysis.

Results for each individual plasma marker are summarized in table e-2A. CK, ferritin, TNF-α, IL-1β, IL-2, IL-8, IL-12p70, IL-4, IL-5, IL-10, and IL-13 were all found to be significantly increased, while IFN-γ was significantly decreased in plasma from patients with ALS compared with controls in both basic and multivariate models. IL-6 showed a similar pattern of increase in the basic model, which was not maintained after adjustment for comorbidities, treatments, and CRP levels.

**Figure 2.**
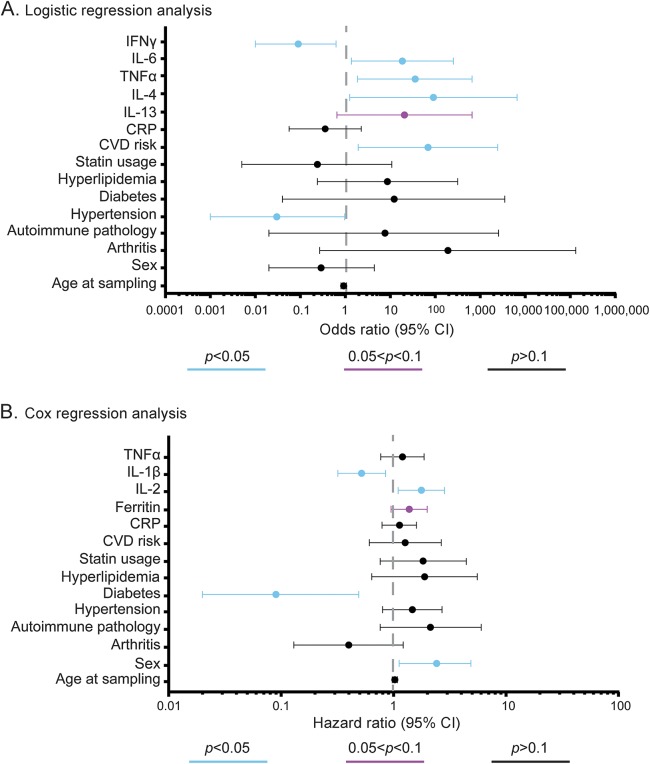
Odds ratios (ORs) from the multivariate logistic regression analysis and hazard ratios (HRs) from the Cox regression analysis (A) OR (filled circle) and relative 95% confidence interval (CI) (whiskers) of plasma markers in amyotrophic lateral sclerosis (ALS) and controls from mutually adjusted multivariate logistic regression models; dashed line represents null hypothesis (no difference between ALS and controls). (B) HR (filled circles) and relative 95% CI (whiskers) of plasma markers estimating risk of death among patients with ALS from mutually adjusted multivariate Cox regression analysis. The dashed line represents null hypothesis (no association with survival). *p* < 0.5, 0.5 < *p* < 0.1, and *p* > 0.1 are shown in blue, mulberry, and black, respectively. CRP = C-reactive protein; CVD = cardiovascular disease; IFN = interferon; IL = interleukin; TNF = tumor necrosis factor.

Mutually adjusted multivariate regression analysis identified only IFN-γ, IL-6, TNF-α, IL-4, and IL-13 as significantly different between ALS cases and controls (table e-2B). Point estimates and relative 95% confidence intervals (CIs) from the mutually adjusted multivariate logistic regression analysis are shown in figure 2A. IFN-γ levels were significantly lower among ALS cases compared to controls (odds ratio [OR] 0.09, 95% CI 0.01–0.62, *p* = 0.02), while IL-6 (OR 18.55, 95% CI 1.37–251.95, *p* = 0.03), TNF-α (OR 35.78, 95% CI 1.86–689.20, *p* = 0.02), IL-4 (OR 90.90, 95% CI 1.25–6,603.62, *p* = 0.04), and IL-13 (OR 20.69, 95% CI 0.65–656.10, *p* = 0.086) were higher in ALS cases compared to controls. There were no significant differences in patients with ALS subgrouped using progression rate at baseline (cutoff at 0.5).

#### Cox regression analysis in patients with ALS.

Examined individually, higher levels of ferritin were found to be associated with poorer survival in ALS in both basic and multivariate models (multivariate hazard ratio [HR] 1.71, 95% CI 1.20–2.44, *p* = 0.003) (table e-2C). Higher IL-2 levels in ALS were associated with poorer survival only in the multivariate model (HR 1.43, 95% CI 1.04–1.98, *p* = 0.03).

Four markers, including ferritin, IL-2, IL-1β, and TNF-α, were further tested in the mutually adjusted multivariate Cox regression analysis and the results are summarized in figure 2B and table e-2D. Higher IL-2 (HR 1.77, 95% CI 1.10–2.84, *p* = 0.02) and ferritin (HR 1.38, 95% CI 0.95–1.99, *p* = 0.09) levels were associated with a shorter survival, while high levels of IL-1β (HR 0.52, 95% CI 0.32–0.85, *p* = 0.009) and the presence of diabetes (HR 0.09, 95% CI 0.02–0.49, *p* = 0.005) were associated with a longer survival.

### Correlation analysis.

We examined the correlation between inflammatory markers and neuromuscular markers as well as ALS disease stages/progression to obtain insights into potential mechanisms underlying ALS pathology and its tissue origin. Patients with higher CK (used as a marker of muscular involvement) levels were found to have higher IL-5 (Spearman ρ: 0.217, *p* = 0.045), while those with higher NfL levels (as a neuronal marker) also had higher TNF-α levels (ρ: 0.264, *p* = 0.033). Patients with higher CRP levels (used as a proxy for general and aspecific inflammatory response) also had higher IL-6 levels (ρ: 0.518, *p* < 0.0001). Conversely, ferritin was found not to be correlated with any of the other markers under investigation. Patients with higher progression rate at baseline had borderline higher ferritin levels (ρ: 0.186, *p* = 0.081) and lower CK levels (ρ: −0.203, *p* = 0.058). Patients with ALS at earlier disease stage (with higher ALSFRS-R score) were reported to have higher CK levels (ρ: 0.401, *p* = 0.0001) and lower CRP (ρ: −0.273, *p* = 0.008), TNF-α (ρ: −0.269, *p* = 0.009), and IL-6 (ρ: −0.217, *p* = 0.001) levels.

### Longitudinal analysis.

Plasma cytokine levels obtained from 59 patients with ALS at baseline (V1) and on their follow-up visits (V2–6) are shown in a box-and-whisker plot (figure e-1). All individual cytokines had comparable median levels of expression in all 6 visits, with the exception of IL-6, which showed a small but significant (*p* = 0.008, Kruskal-Wallis test) increase at V6 (median [IQR] levels: 0.81 [0.56, 1.03], n = 21) compared to V1 (0.44 [0.30, 0.66], n = 58; adjusted for multiple comparison, *p* = 0.002). The median ALSFRS-R scores (IQR) at V1 and V6 were 39 (34.5, 43.0) and at 32.5 (21.5, 36.0), respectively. Data from the baseline measurements in all ALS cases under investigation (n = 95) and in controls (n = 88) are also provided in figure e-1 as references.

**Figure 3.**
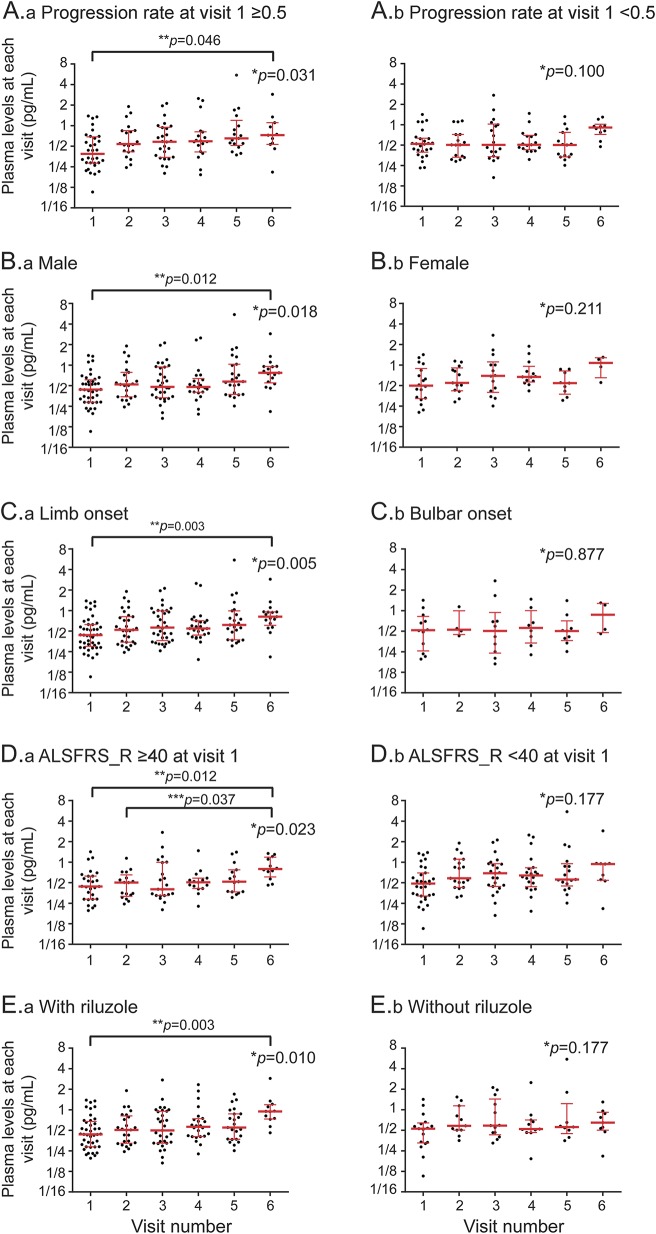
Longitudinal expression levels of interleukin (IL)–6 for patients with amyotrophic lateral sclerosis (ALS) The scatterplots show IL-6 plasma levels (black dots) obtained at each follow-up time point from the 59 patients with ALS included in the longitudinal cohort. Median and quartile ranges at each visit are presented with red bars. Patients were subgrouped according to (A.a, A.b) progression rate calculated at baseline (with a cutoff value of 0.5); (B.a, B.b) sex; (C.a, C.b) site of disease onset; (D.a, D.b) ALS Functional Rating Scale–Revised (ALSFRS-R) score at V1 (cutoff value of 40); and (E.a, E.b) whether they were on riluzole or not. *p Value for Kruskal-Wallis test examining difference between all visits; **p value adjusted for multiple comparison between V1 and V6; ***p value adjusted for multiple comparison between V2 (D only) and V6.

Figure 3 displays the scatterplots of longitudinal IL-6 levels in plasma from patients with ALS subgrouped according to progression rate at baseline, sex, site of onset, ALSFRS-R score, and the use of riluzole. A mild but significant increase of IL-6 plasma levels towards the end of follow-up (V6) is demonstrated in slow progressors, male, limb onset, less functional impairment at V1, and ALS cases treated with riluzole. In the longitudinal cohort, patients treated with riluzole and those not on treatment (n = 42/17) had comparable features such as age at onset, sex, age/progression rate/ALSFRS-R score at baseline, disease duration between onset and diagnosis, and disease duration between diagnosis and baseline.

We examined the correlation between cytokines and the progression of disease in patients with ALS during the follow-up period (intervisit progression: ΔALSFRS-R and the slope of ALSFRS-R); no meaningful associations were found between the intervisit progression and the plasma cytokine levels at the earlier visit in 212 consecutive visit pairs obtained from our longitudinal cohort of 59 patients with ALS. No association was observed between CRP, CK, and ferritin and the V1–V2 intervisit progression.

## DISCUSSION

The results of our study show a dichotomy in the pattern of cytokine regulation in blood from patients with ALS, with a broad but not uniform upregulation of TNF-α, IL-1β, IL-2, IL-8, IL-12p70, IL-4, IL-5, IL-10, and IL-13, in line with previous studies,^18,19^ and the downregulation of IFN-γ. When the combined effects of all markers are accounted for, IL-6, TNF-α, and IFN-γ emerge as strong and coherent disease signals, as already shown in a range of other pathologic conditions.^20^

All cytokines under investigation in this study show stable levels of expression throughout the follow-up period, except for IL-6, which undergoes a late-stage upregulation. This preliminary observation needs to be further validated, considering that IL-6 has recently been established as a therapeutic target in ALS and a phase II clinical trial is planned.^21^ The strong proinflammatory effect of TNF-α has previously been associated with late-stage ALS, when neuroinflammation is most detrimental to motor neurons.^20^ Strong evidence supports TNF-α-induced oxidative damage to actin, resulting in the collapse of growth cones and neurite retraction.^22^ In line with these observations, the positive correlation between TNF-α and plasma NfL observed in this study supports a role for TNF-α in neuroaxonal destruction, with downstream NfL release in biological fluids. Our finding of a significant reduction of IFN-γ compared to controls after adjustment for comorbidities and treatments is intriguing and in contrast with previous data from smaller case-control studies showing elevated IFN-γ levels in postmortem spinal cord, CSF, and serum from patients with ALS.^18,23^ Our observation may be explained by the relocation of IFN-γ-secreting Th1 cells into the CNS^24^ or by the upregulation of IFN-γ receptor in the CNS in response to the high levels of TNF-α and IL-1.^25,26^

Of the activated inflammatory markers in our study, IL-6, IL-8, TNF-α, IL-10, IL-4, and IL-13 can be produced physiologically by contracting muscle fibers and can be overexpressed during strength training.^27^ It is possible that hyperexcitable muscles prone to fasciculation and atrophy in ALS may also cause overexpression of these markers. In rodent models of ALS, neuromuscular junction destruction and distal axonopathy precede motor neuron loss^28^; the early accumulation of macrophages expressing CD11b and CD68 in axons^29^ and of other immunologic factors originating from the muscle may play an active role in motor neuron degeneration by inhibition of neurite outgrowth.

IL-6 promotes glucose uptake and fatty acid oxidation–induced lipolysis and regulates muscle–adipose tissue crosstalk.^30^ The systemic immune response we observe in ALS may be linked to the state of hypermetabolism and of lipid dysregulation now widely recognized in ALS.^31^ Hence, the observed late IL-6 regulation may reflect the increasing metabolic imbalance associated with the disease process in ALS. We also report how the expression of CK, a known marker for muscular damage, and of IL-5, which is central to B-cell immunity and eosinophil activation, change synergistically. IL-5 and other cytokines driving the more benign form of inflammation defined as Th2 have not been reported to change in plasma expression after exercise.^27^ Since high CK plasma levels are also associated with better neurologic function (higher ALSFRS-R score) and slower progression rate in our patients with ALS, the correlation between CK and IL-5 expression may be indicative of neurorestorative mechanisms rather than being a measure of neuromuscular dysfunction. The speculative nature of these observations will need further studies to improve our understanding of the pathologic processes linked to ALS.

Our panel of inflammatory markers and NfL have both been reported as differentially regulated in blood from patients with ALS and to have a prognostic value with regard to the rate of disease progression. However, the reported changes of circulating inflammatory markers throughout the disease course may be a more representative measure of the disease burden in a neuromuscular disorder like ALS, which engenders destruction of motor areas in the CNS and muscle functional derangement by denervation. Neurofilaments release in biological fluids relates only to neuroaxonal damage and not to the wider neuromuscular pathology observed in ALS. Hence, NfL and the reported inflammatory markers should be considered as complementary signals within an improved panel of disease biomarkers for ALS.

Among the activated markers in our study, IL-2 and ferritin are risk factors for survival. IL-2 has not been directly linked to ALS as a biomarker, nor has it been found to have a direct pathogenic role in the disease. IL-2 may modulate the disease process by induction of Treg or by activation of natural killer (NK) cells known to be cytotoxic for a wide range of neurons. The reported increase of NK and CD8+ T cells in the blood of patients with ALS supports not only the important contribution of the innate immune system in the development of ALS, but also a potential role for IL-2.^9,32^ The interplay among IL-2, Treg, NK, and cytotoxic T cells is at the center of intense research into potential immunomodulatory therapeutic strategies in ALS.

Our data on ferritin upregulation in blood, its prognostic significance, and positive correlation with PRB in patients with ALS supports previous findings,^5,33^ even though a protective effect was reported in a small sample–sized predictive model.^34^ Ferritin is able to sequester iron and reduce the amount of iron available for reactive oxygen species, thus acting as a defense mechanism against oxidative stress.^35^ It has been shown that TNF-α and IL-1α can regulate ferritin transcriptionally,^36^ while TNF-α, IL-1β, and IL-6 exert the same function post-transcriptionally.^37,38^ These observations corroborate the marked systemic upregulation of these cytokines and the prognostic effect of ferritin observed in our study. With regard to changes that may be associated with a better prognostic outlook, we have also observed a protective effect of diabetes for the survival of our patients with ALS (figure 2B), which is in line with a recent population study from Danish cohorts.^39^

It has previously been shown that CSF hosts relevant components of ALS immunoreactivity,^19^ including the regulation of the proinflammatory and neutrophil activator IL-8 linked to shorter disease duration and of the monocyte chemoattractant protein-1 (MCP-1) associated with prolonged survival.^34^ CSF is unanimously considered the most reliable source of biological signals of neurodegeneration for its anatomical contiguity to affected nervous tissues; it is naturally enriched of by-products of neuronal destruction or remodeling. Recently, combined CSF and plasma inflammatory markers have been proposed as an orthogonal biomarker to model prognosis in ALS, including plasma IFN-γ-induced protein 10, IL-5, and L-ferritin, as well as CSF IL-8, MCP-1, and IFN-γ plasma/CSF ratio.^34^ However, immunologic signals and other tissue-specific signals arising from the neuromuscular pathology seen in ALS may be underrepresented in CSF. Lumbar punctures are also impractical when serial CSF sampling is needed for longitudinal biomarker studies, particularly in patients with advanced ALS. Peripheral blood represents a complex but more accessible alternative to CSF for long-term immune monitoring of ALS^40^ and the most appropriate matrix to measure systemic changes linked to the development of neurodegeneration.

The composite inflammatory response we report in this article, with the variable regulation of TNF-α, IL-6, and IFN-γ, the rising levels of IL-6 with disease progression, and the prognostic values of IL-2/ferritin with regard to survival is an additional tool for better comprehensive disease stratification in ALS. This preliminary observation requires further validation in larger and independent ALS cohorts. A better understanding of the regulation of circulating immunologic factors in patients with ALS has several advantages and could be used (1) to increase the diagnostic potency of existing panels of neurochemical biomarkers, (2) for the prognostic stratification of the disease, and (3) to assess treatment response in clinical trials, particularly if immunomodulatory strategies are involved. The adjustment for factors involved in the systemic inflammatory response in our analyses adds to the specificity of the ALS immune response. The proinflammatory or neuroprotective effect of cytokines is likely to depend on the disease stage and the biological microenvironment they are embedded in. Only the combined monitoring of the expression of these immunologic markers and of other clinical and biological measures of disease progression and of treatment response can establish inflammatory markers as useful disease monitoring tools in ALS.

## Supplementary Material

Data Supplement

## GLOSSARY

ALS: amyotrophic lateral sclerosis
ALSFRS-R: ALS Functional Rating Scale–Revised
CI: confidence interval
CK: creatine kinase
CRP: C-reactive protein
CVD: cardiovascular disease
FTD: frontotemporal dementia
HR: hazard ratio
IFN: interferon
IL: interleukin
IQR: interquartile range
LOD: limit of detection
MCP-1: monocyte chemoattractant protein–1
NfL: neurofilament light chain
NK: natural killer
OR: odds ratio
PCA: principal component analysis
PRB: progression rate calculated at baseline
TNF: tumor necrosis factor
Treg: regulatory T cells
