# Supplementary material for: Systemic inflammatory response and neuromuscular involvement in amyotrophic lateral sclerosis
Source: Neurol Neuroimmunol Neuroinflamm. 2016 Jun 1;3(4):e244. doi: 10.1212/NXI.0000000000000244 (PMC4897985; doi:10.1212/NXI.0000000000000244)
Supplement: Data Supplement [file supp_3_4_e244__index.html]

Data Supplement 

# Systemic inflammatory response and neuromuscular involvement in amyotrophic lateral sclerosis

## Data Supplement

**Files in this Data Supplement:**

- Figure e-1 - PDF
- Table e-1 - PDF
- Table e-2 - PDF
