# Supplementary material for: Systemic inflammatory response and neuromuscular involvement in amyotrophic lateral sclerosis
Source: Neurol Neuroimmunol Neuroinflamm. 2016 Jun 1;3(4):e244. doi: 10.1212/NXI.0000000000000244 (PMC4897985; doi:10.1212/NXI.0000000000000244)
Supplement: Data Supplement [file supp_3.4.e244_fig_e1.pdf]

Figure e-1

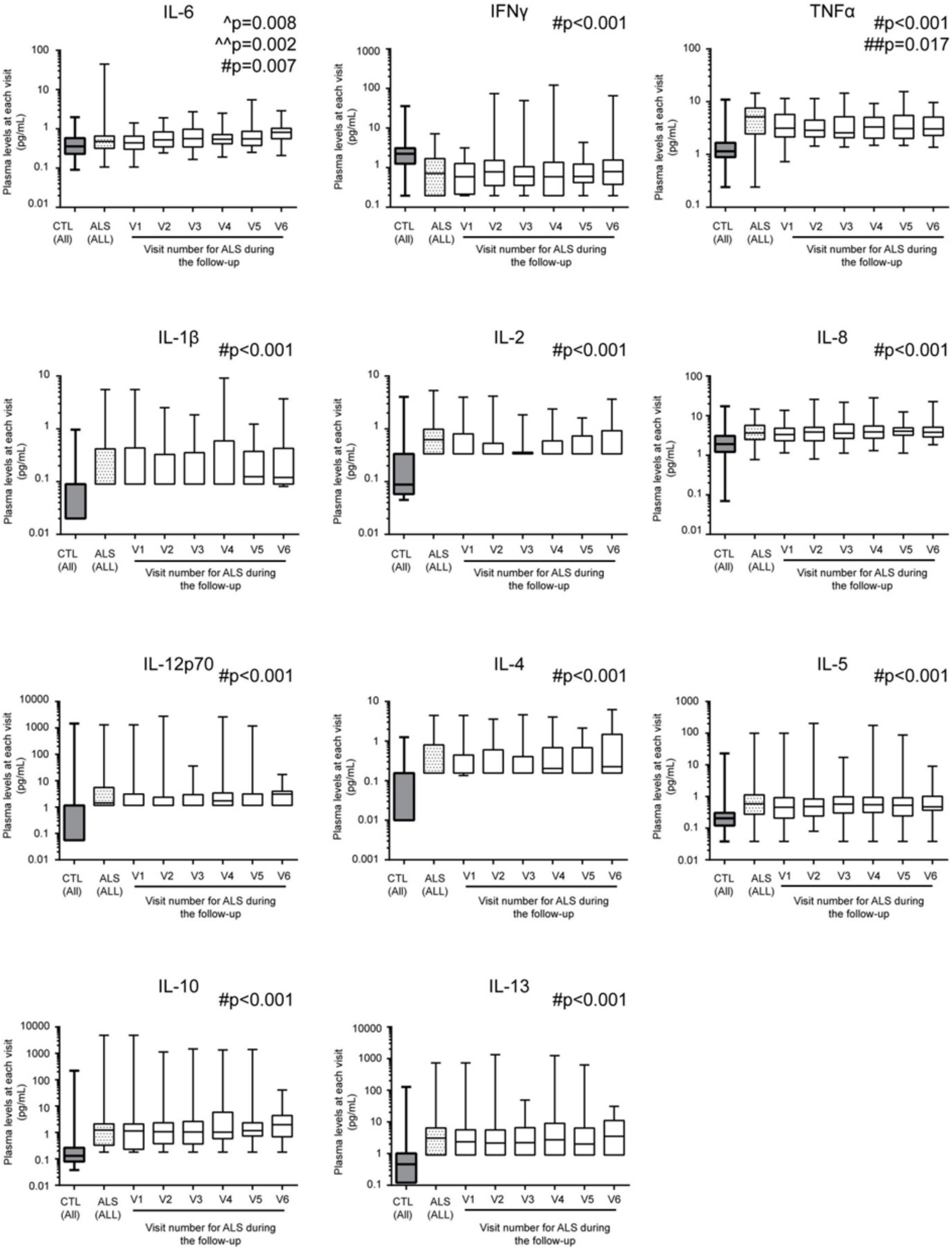
IL-6
^p=0.008
^^p=0.002
#p=0.007
IFNγ
#p<0.001
TNFα
#p<0.001
##p=0.017
IL-1β
#p<0.001
IL-2
#p<0.001
IL-8
#p<0.001
IL-12p70
#p<0.001
IL-4
#p<0.001
IL-5
#p<0.001
IL-10
#p<0.001
IL-13
#p<0.001
Plasma levels at each visit (pg/mL)
CTL (All)
ALS (ALL)
V1
V2
V3
V4
V5
V6
Visit number for ALS during the follow-up

Figure e-1. Plasma cytokine levels of ALS patients at each visit in the longitudinal and case-control studies.

The box-and-whisker plots show the expression of the 11 cytokines obtained at baseline (V1) and during the 15-month follow-up period (V2-V6) from 59 ALS cases (clear box) who underwent serial sampling. The boxes represent the Median (IQR) levels at each visit and the whiskers indicate the ranges. Only IL-6 showed a small but significant increase in expression between V1 and V6. Expression data from all the controls (filled box) and the ALS patients (dotted box) are also presented for reference. ^P-value: Kruskal-Wallis test examining changes of IL-6 expression between visits; ^^p-value adjusted for multiple comparison between V1 and V6; #p-value: Mann-Whitney test examining changes in IL-6 expression between all controls and all ALS; ##p-value: Mann-Whitney test examining changes of TNFα expression between all ALS and the 59 ALS included in the longitudinal cohort; not significant p-values are not reported.
