# Supplementary material for: Systemic inflammatory response and neuromuscular involvement in amyotrophic lateral sclerosis
Source: Neurol Neuroimmunol Neuroinflamm. 2016 Jun 1;3(4):e244. doi: 10.1212/NXI.0000000000000244 (PMC4897985; doi:10.1212/NXI.0000000000000244)
Supplement: Data Supplement [file supp_3.4.e244_table_e1.pdf]

Table e-1. Summary of cut-off used to determine tertiles in Logistic regression analysis and Cos regression analysis.

**Table in the main text**	**Table e-2A**		**Table e-2B**		**Table e-2C & Table e-2D**	
Defined by	All subjects		Case and Controls without any missing data		ALS only	
Cut-off for 1st and 2nd tertiles (A) & 2nd and 3rd tertiles (B)	**A**	**B**	**A**	**B**	**A**	**B**
**CRP (mg/L)**	2.000	5.000	2.000	4.500	2.000	6.000
**CK (U/L)**	98.655	165.017	91.662	169.000	127.988	273.006
**Ferritin (µg/L)**	95.000	177.339	97.324	159.676	118.652	235.670
**IL-6 (pg/mL)**	0.321	0.538	0.317	0.545	0.362	0.569
**IFNγ (pg/mL)**	0.915	2.175	0.843	2.218	0.397	1.304
**TNFα (pg/mL)**	1.405	5.041	1.185	2.544	3.138	6.496
**IL-1β (pg/mL)**	0.038	0.090	0.020	0.090	0.090	0.472
**IL-2 (pg/mL)**	0.189	0.356	0.101	0.335	0.335	0.920
**IL-8 (pg/mL)**	2.010	3.620	1.752	3.189	2.931	4.301
**IL-12p70 (pg/mL)**	0.110	1.451	0.069	1.150	1.150	5.083
**IL-4 (pg/mL)**	0.012	0.166	0.010	0.155	0.155	0.801
**IL-5 (pg/mL)**	0.197	0.579	0.165	0.406	0.373	0.946
**IL-10 (pg/mL)**	0.180	1.157	0.158	0.745	0.638	1.658
**IL-13 (pg/mL)**	0.900	2.945	0.595	1.253	1.039	4.853

Cut-off values used for tertile transformation in the logistic regression analysis and Cox regression analysis.
