# Supplementary material for: Systemic inflammatory response and neuromuscular involvement in amyotrophic lateral sclerosis
Source: Neurol Neuroimmunol Neuroinflamm. 2016 Jun 1;3(4):e244. doi: 10.1212/NXI.0000000000000244 (PMC4897985; doi:10.1212/NXI.0000000000000244)
Supplement: Data Supplement [file supp_3.4.e244_table_e2.pdf]

Table e-2. Summary of logistic regression analysis for each plasma marker.

**Table e-2A**	***ALS vs Controls:*** **OR and relative 95% CI of *individual* plasma markers, with and without further adjustment for clinical variables.**			
	**Basic model[a]**		**Multivariate model[b]**	
**Markers**	**OR (95%CI)**	**p-value**	**OR (95%CI)**	**p-value**
**CK**	2.41 (1.54, 3.78)	<0.001	3.03 (1.80, 5.10)	<0.001
**Ferritin**	2.10 (1.37, 3.23)	0.001	2.17 (1.35, 3.47)	0.001
**CRP**	1.26 (0.79, 2.01)	0.33	N/A	N/A
**IL-6**	1.34 (0.89, 2.02)	0.16	1.28 (0.80, 2.04)	0.31
**TNFα**	8.00 (4.33, 14.76)	<0.001	7.24 (3.81, 13.77)	<0.001
**IL-1β**	7.41 (4.01, 13.69)	<0.001	6.60 (3.51, 12.39)	<0.001
**IL-8**	3.31 (2.08, 5.26)	<0.001	3.42 (2.03, 5.74)	<0.001
**IFNγ**	0.20 (0.12, 0.34)	<0.001	0.21 (0.12, 0.37)	<0.001
**IL-2**	15.49 (7.12, 33.68)	<0.001	14.22 (6.32, 32.01)	<0.001
**IL-12p70**	12.51 (7.12, 33.68)	<0.001	11.41 (5.32, 24.48)	<0.001
**IL-4**	9.74 (5.02, 18.90)	<0.001	10.96 (5.19, 23.18)	<0.001
**IL-5**	3.49 (2.17, 5.61)	<0.001	3.59 (2.14, 6.03)	<0.001
**IL-13**	11.38 (5.52, 23.46)	<0.001	11.05 (5.15, 23.69)	<0.001
**IL-10**	10.67 (5.27, 21.59)	<0.001	10.19 (4.84, 21.48)	<0.001
**Table e-2B**	***ALS vs Controls*** **OR and relative 95% CI of *mutually-adjusted* plasma markers in a basic and a multivariate model.**			

	Basic model[a]		Multivariate model[b]	
**Markers**	**OR (95% CI)**	**p-value**	**OR (95% CI)**	**p-value**
**Age at sampling, y**	1.05 (0.99, 1.12)	0.11	0.92 (0.80, 1.06)	0.26
**Gender[c]**	0.17 (0.03, 0.86)	0.03	0.29 (0.02, 4.39)	0.37
**CK**	1.36 (0.54, 3.43)	0.51	0.88 (0.23, 3.33)	0.85
**Ferritin**	1.74 (0.70, 4.33)	0.23	0.61 (0.13, 2.81)	0.53
**CRP**	0.54 (0.14, 2.13)	0.38	0.34 (0.05, 2.15)	0.25
**IL-6**	5.53 (1.31, 23.30)	0.02	18.55 (1.37, 251.95)	0.03
**TNFα**	5.05 (0.93, 27.59)	0.06	35.78 (1.86, 689.20)	0.02
**IL-1β**	1.37 (0.24, 7.78)	0.72	0.79 (0.06, 10.11)	0.86
**IL-8**	0.63 (0.28, 1.73)	0.37	0.34 (0.07, 1.78)	0.20
**IFNγ**	0.14 (0.04, 0.55)	0.005	0.09 (0.01, 0.62)	0.02
**IL-2**	1.96 (0.16, 24.74)	0.61	0.23 (0.01, 8.41)	0.43
**IL-12p70**	0.60 (0.04, 10.22)	0.72	0.06 (0.001, 2.79)	0.15
**IL-4**	2.05 (0.40, 10.52)	0.39	90.90 (1.25, 6603.62)	0.04
**IL-5**	1.27 (0.33, 4.84)	0.73	1.64 (0.31, 8.73)	0.57
**IL-13**	4.88 (0.52, 45.99)	0.17	20.69 (0.65, 656.10)	0.086
**IL-10**	1.001 (0.99, 1.02)	0.86	1.005 (0.98, 1.03)	0.74
**Arthritis[d]**	N/A		190.63 (0.27, 133073.73)	0.12

**Autoimmune pathology**[d]			7.61 (0.02, 2561.16)	0.49
**Hypertension**[d]			0.03 (0.001, 0.97)	0.048
**Diabetes**[d]			12.18 (0.04, 3510.54)	0.39
**Hyperlipidaemia**[d]			8.65 (0.24, 314.33)	0.24
**Statin Usage**[d]			0.24 (0.005, 10.76)	0.46
**CVD Risk**			68.82 (1.94, 2444.97)	0.02
**Table e-2C**	**HR and relative 95% CI of *individual* plasma markers with and without further adjustment for clinical variables to evaluate survival *among ALS patients.***			
	**Basic model**[a]		**Multivariate model**[b]	
**Markers**	**HR (95% CI)**	**p-value**	**HR (95% CI)**	**p-value**
**CK**	0.95 (0.70, 1.29)	0.74	0.92 (0.67, 1.26)	0.59
**Ferritin**	1.74 (1.26, 2.41)	0.001	1.71 (1.20, 2.44)	0.003
**CRP**	1.19 (0.88, 1.61)	0.26	N/A	N/A
**IL-6**	1.04 (0.77, 1.40)	0.82	1.02 (0.73, 1.42)	0.92
**TNFα**	1.26 (0.94, 1.71)	0.13	1.31 (0.94, 1.81)	0.11
**IL-1β**	0.81 (0.60, 1.11)	0.18	0.70 (0.48, 1.03)	0.07
**IL-8**	1.07 (0.80, 1.45)	0.65	1.18 (0.84, 1.66)	0.35
**IFNγ**	1.05 (0.78, 1.42)	0.74	1.01 (0.73, 1.39)	0.97

**IL-2**	1.29 (0.96, 1.74)	0.09	1.43 (1.04, 1.98)	0.03
**IL-12p70**	1.07 (0.80, 1.42)	0.66	1.09 (0.80, 1.48)	0.60
**IL-4**	0.94 (0.69, 1.27)	0.68	0.98 (0.68, 1.40)	0.90
**IL-5**	0.95 (0.72, 1.25)	0.71	0.89 (0.65, 1.22)	0.47
**IL-13**	1.01 (0.75, 1.36)	0.96	1.00 (0.72, 1.38)	0.99
**IL-10**	1.06 (0.80, 1.41)	0.69	1.05 (0.77, 1.43)	0.77
**Table e-2D**	**HR and relative 95% CI of *__mutually-adjusted__* plasma markers in a basic and a multivariate model to evaluate survival *__among ALS patients.__***			
	**Basic model**[a]		**Multivariate model**[b]	
**Markers**	**HR (95% CI)**	**p-value**	**HR (95% CI)**	**p-value**
**Age at sampling, y**	1.05 (1.02, 1.08)	0.002	1.03 (0.97, 1.08)	0.37
**Gender**[c]	1.77 (1.01, 3.12)	0.047	2.42 (1.12, 4.88)	0.02
**Ferritin**	1.46 (1.04, 2.04)	0.03	1.38 (0.95, 1.99)	0.09
**IL2**	1.61 (1.05, 2.47)	0.03	1.77 (1.10, 2.84)	0.02
**IL1β**	0.55 (0.35, 0.85)	0.01	0.52 (0.32, 0.85)	0.009
**TNFα**	1.18 (0.77, 1.81)	0.45	1.20 (0.77, 1.87)	0.42
**Hypertension**[d]	N/A		1.47 (0.80, 2.70)	0.21
**Diabetes**[d]			0.09 (0.02, 0.49)	0.005
**Hyperlipidaemia**[d]			1.89 (0.64, 5.55)	0.25
**Statin usage**[d]			1.83 (0.76,4.43)	0.18

**CVD risk**		1.27 (0.61, 2.66)	0.52
**CRP**		1.13 (0.79, 1.60)	0.51
**Arthritis**[d]		0.40 (0.13, 1.22)	0.11
**Autoimmune pathology**[d]		2.13 (0.76, 6.03)	0.15

Abbreviations: OR, odds ratios; CI, confidence intervals; HR, hazard ratios; N/A, not applicable. [a], Basic model: adjusted for gender and age at sampling; [b], Multivariate model: adjusted for gender, age at sampling, CRP, hypertension, diabetes, hyperlipidaemia, statin usage, CVD risk, arthritis and autoimmune pathology; [c], reference group: male; [d], reference group: without the disease/treatment.
